# Reading the tea leaves: Dead transposon copies reveal novel host and transposon biology

**DOI:** 10.1371/journal.pbio.2005470

**Published:** 2018-03-05

**Authors:** Richard N. McLaughlin

**Affiliations:** Pacific Northwest Research Institute, Seattle, Washington, United States of America

## Abstract

Transposable elements comprise a huge portion of most animal genomes. Unlike many pathogens, these elements leave a mark of their impact via their insertion into host genomes. With proper teasing, these sequences can relay information about the evolutionary history of transposons and their hosts. In a new publication, Larson and colleagues describe a previously unappreciated density of long interspersed element-1 (LINE-1) sequences that have been spliced (LINE-1 and other reverse transcribing elements are necessarily intronless). They provide data to suggest that the retention of these potentially deleterious splice sites in LINE-1 results from the sites’ overlap with an important transcription factor binding site. These spliced LINE-1s (i.e., spliced integrated retrotransposed elements [SpiREs]) lose their ability to replicate, suggesting they are evolutionary dead ends. However, the lethality of this splicing could be an efficient means of blocking continued replication of LINE-1. In this way, the record of inactive LINE-1 sequences in the human genome revealed a new, though infrequent, event in the LINE-1 replication cycle and motivates future studies to test whether splicing might be another weapon in the anti-LINE-1 arsenal of host genomes.

A piddling portion of most animal genomes encodes proteins. Instead, much of the DNA real estate in the genome of humans and other animals was built by the replication of selfish genetic elements. Selfish elements are stretches of DNA that encode the information necessary to replicate their own sequence in the context of a host cell [1, 2]. By definition, these elements provide no specific utility to the host cell; however, it is becoming increasingly clear that sometimes these elements can be co-opted into roles that may benefit the host. Cellular life plays host to a dazzling diversity of these selfish elements, including one group called transposons, characterized by their ability to move from one location in a genome to another. Some cut and paste themselves within genomes. Others mobilize through copy-and-paste mechanisms, leaving the parent sequence in place and inserting a new copy elsewhere in the genome.

In humans, a single group of copy-and-paste transposons called the long interspersed elements-1 (LINE-1s) is responsible for most transposition events (the moving of a sequence from one genomic location to another; Box 1, Fig 1). The long-standing presence of LINE-1 in the genomes of humans and their ancestors is starkly evident in the massive portion of the human genome derived from these transposons. Our ability to resolve homology deteriorates with aging of a transposon insertion, but about 17% of the human genome can be confidently assigned as LINE-1 sequence, and more than 10% comes from other transposons mobilized by LINE-1 [3]. By some estimates, the replication of LINE-1 and other transposons together may have contributed over two-thirds of the human genome [4]. Successful replication of these elements requires integration into the germline genome but also leads to the “bloated” size of the human and other animal genomes. However, these sequences, mostly degraded and inactive, provide a sort of fossil record of the transposable elements in the genome of each species. Indeed, researchers have tracked the activity over time of different transposons in the genome of various organisms using these transposon fossils [5]. Intriguingly, these sequences should also contain information about the adaptation of transposons over evolutionary timescales.

**Box 1. Replication of long interspersed element-1 (LINE-1) retroelements in the human genome**

Retroelement-derived sequences comprise over 50% of the human genome [3, 4, 6]. The most prevalent of these, LINE-1 elements, contain an internal promoter in their 5′-untranslated region (UTR), 2 open reading frames (ORFs), and a short 3′-UTR containing the polyadenylation site used for reverse transcription [7–9]. ORF1 encodes the ORF1p protein, which contains a coiled-coil protein interaction domain and a nucleic acid-binding domain with chaperone activity [10]. ORF2 encodes the ORF2p protein with endonuclease, reverse transcriptase, and a cysteine-rich region [11, 12]. As part of their copy-and-paste replication, termed retrotransposition, LINE-1s are transcribed, and the resulting RNA is reverse transcribed (by LINE-1-encoded proteins) into a new DNA copy of the element (Fig 1). To increase in copy number, LINE-1 must replicate in the germline or early embryo in order to pass additional copies to host offspring. Host factors act at various steps in the lifecycle of LINE-1 to block this replication. These factors impose their restriction through various means, including transcriptional repression, degradation, and mutation of the various nucleic acid intermediates of retrotransposition [13]. LINE-1 and other retroelements have been shown to cause a variety of diseases in humans by interrupting genes, acting as regulatory elements that drive abnormal expression, or inducing aberrant ectopic recombination [14–16]. Despite constituting a large fraction of the human genome, only a very small fraction of retroelements are competent to mobilize, and an even smaller fraction comprise most of the retrotransposition activity in the human genome. For instance, there are no active endogenous retroviruses in the human genome. Similarly, only 3,000–5,000 of the estimated 500,000 LINE-1 sequences in the human genome are full length. Of these, 80–100 are estimated to be retrotransposition competent, and only 6 “hot” LINE-1s are responsible for 84% of the retrotransposition events in human cells [17].

**Fig 1:**
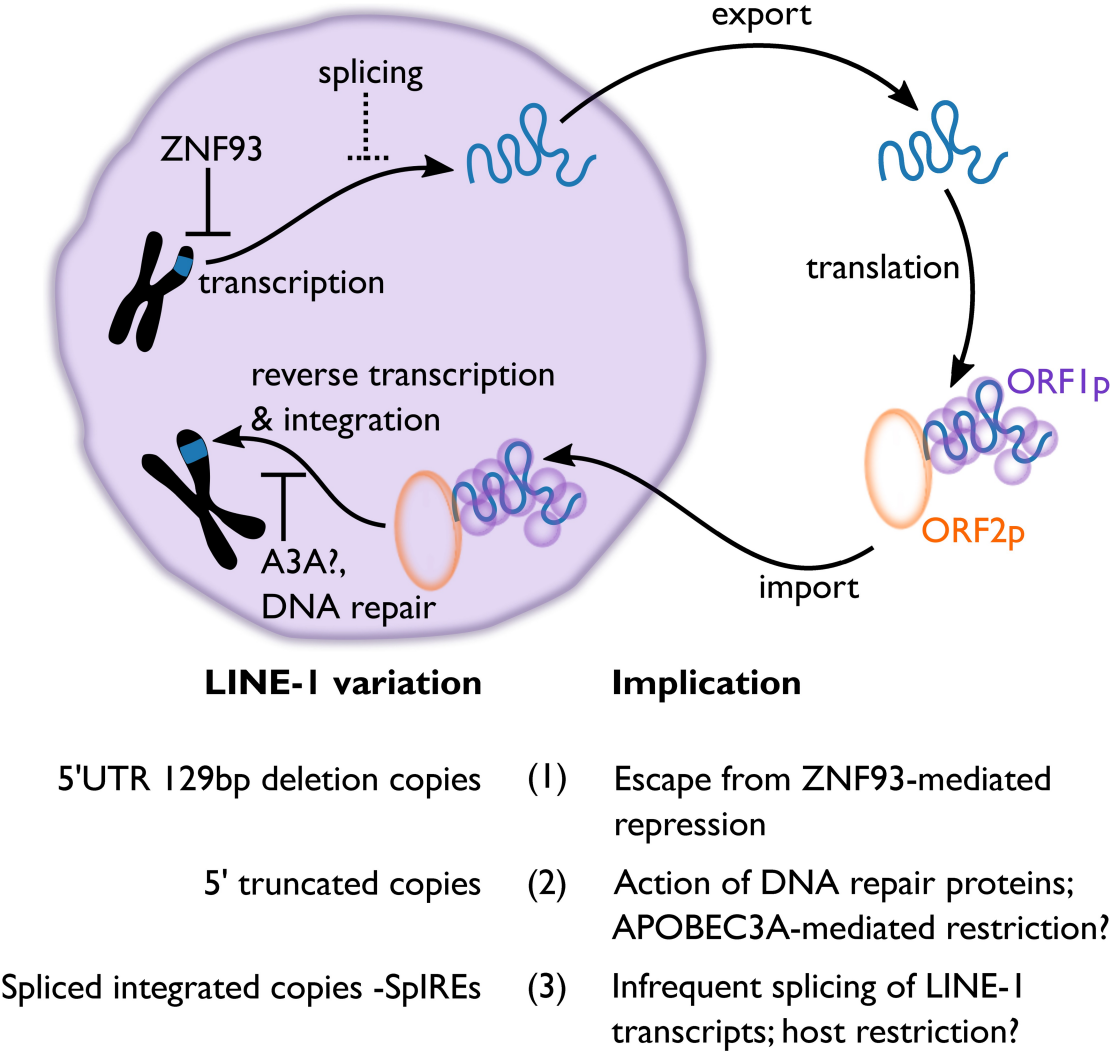
The long interspersed element-1 (LINE-1) lifecycle and lessons from ancient LINE-1 sequences. LINE-1 (long interspersed element-1) retrotransposons replicate using an RNA intermediate that encodes 2 proteins: ORF1p binds LINE-1 RNA, and ORF2p reverse transcribes and integrates that RNA to create a new LINE-1 copy (see Box 1). As a part of this replication, LINE-1 sequences must insert into the host genome. A genome’s inventory of these integrated sequences can be used to understand specific aspects of host and LINE-1 biology. For example, (1) LINE-1 sequences in the human genome are polymorphic for a 129-bp deletion in their 5′-untranslated region (UTR). This deletion allows LINE-1 sequences to evade the repressive effects of a DNA-binding protein (ZNF93) that initiates transcriptional silencing [18]; (2) most of the LINE-1 sequences in the human genome are 5′ truncated. This may be due to the action of APOBEC3A, which deaminates single-stranded DNA at the site of LINE-1 insertion [19], or DNA repair proteins [20, 21]; (3) about 2% of all full-length LINE-1 sequences in the genome have been spliced, as shown by new research from Larson and colleagues [22]. This splicing inactivates the new LINE-1 copies, but retention of the splice donor site preserves a transcription factor binding site that drives efficient LINE-1 transcription. It is unclear whether host cells employ splicing to actively block LINE-1 replication. Abbreviations: SpIRE, spliced integrated retrotransposed element; UTR, untranslated region.

When we think about a transposon or a virus, we envision the ordered, logical lifecycle schematized in multitudinous reviews and textbooks. Biology is on average neat and tidy, but there is a possibility for lots of unusual molecular events to happen—truncated mRNA pieces make protein, spliced RNAs make variant proteins, introns remain unspliced, host proteins introduce mutations into DNA and RNA, etc. Most of these events are relatively rare and, as such, hard to measure in real time. However, the thousands of transposon sequences in a genome provide a historical record of any variant that makes it to the step of integration. Frank Jacobs and colleagues previously revealed that one such variant, namely the deletion of a 129-bp region of LINE-1 that comprises the binding site for a host restriction factor called ZNF93, could provide a substantial benefit to LINE-1 in evading ZNF93-mediated transcriptional repression (Fig 1) [18]. The fossil record of LINE-1 activity in the human genome shows that elements with this deletion remain active in humans. In this way, the genome shows us a history of host restriction and subsequent evasion of LINE-1 in humans and their ancestors. In this issue of *PLOS Biology*, Larson and colleagues show that the LINE-1 fossil record can also elucidate a poorly understood aspect of LINE-1 biology—namely, that some LINE-1 transcripts may be subject to splicing [22].

Previous work from Victoria Belancio and colleagues demonstrated that some LINE-1s contain apparent splice acceptor and splice donor sites [23–25]. Further, evidence of splicing of LINE-1 was found in the presence of several integrated LINE-1 sequences that had been spliced. In new work, Peter Larson and colleagues tackle the intriguing question of why LINE-1 would retain splice acceptor and splice donor sites that could excise necessary portions of the element’s sequence. This splicing would presumably block the ability of descendant insertions to replicate. Larson et al. acronymize these integrated LINE-1 sequences that have been subject to splicing as SpIREs (spliced integrated retrotransposed elements) and present a fascinating hypothesis for the retention of these apparently disrupting sequence features over long evolutionary time.

One reason a seemingly deleterious sequence or trait may be retained in biology is that the deleterious consequences (as measured in the lab) do not manifest in nature. One could certainly imagine that LINE-1 sequences are spliced at a low enough rate that splicing presents no real detriment to the elements. Alternatively, as proposed by Larson et al., a deleterious sequence could be retained because in another context that same sequence provides an outweighing benefit. Here, the authors show that the splice donor site in one of the SpIRE forms overlaps precisely with the binding site of a transcription factor, RUNX3, which drives transcription of LINE-1—an apparently beneficial function [26]. In this way, increasing transcription via RUNX3 binding requires the trade-off of retaining a splice site. If this splice site were used efficiently (it seems not to be in extant LINE-1s) to the point that the detriment of splicing surpassed the benefit of RUNX3 binding-mediated transcription, one would assume that this sequence would be purged from active LINE-1 sequences over time. Further, there could be ways to compensate for the negative impact of splicing. Perhaps ancestral LINE-1s were subject to more efficient, restrictive splicing and sampled some other variant that formed a secondary structure or recruited a protein to obstruct the splice site.

Larson and colleagues proceed beyond a description of SpIREs. They use molecular biology to better understand the consequences of splicing to LINE-1s, in essence trying to mechanistically understand the historical events recorded by the human genome. First, the researchers describe a previously unrecognized SpIRE that is 10 times more abundant in the human genome than previously described SpIREs. They proceed to show that the transcripts from full-length LINE-1 sequences are spliced at very low levels; the vast majority of transcript remains unspliced, and some SpIREs that can be found in the human genome are not produced in measurable quantities using in vitro transcription assays. The clear next question is whether these spliced forms of the LINE-1 sequence are capable of retrotransposition or whether these sequences may be evolutionary dead ends for LINE-1.

The authors venture to measure the inherent transcription levels of various SpIREs using constructs in which the 5′-UTRs of spliced and unspliced LINE-1s were cloned upstream of a reporter gene; normally, the LINE-1 5′-UTR encodes promoter activity sufficient to drive transcription. While the full-length, unspliced UTR efficiently drives transcription, the spliced sequence drives about 30-fold less transcription than the unspliced version. Last, the authors show that SpIREs are severely impaired in their ability to retrotranspose. One SpIRE that transcribes at a low level compared to unspliced LINE-1s can still retrotranspose, but only at a similarly low level. When driven with a strong promoter, however, this SpIRE replicates at about half the level of the unspliced sequence, suggesting the primary block to this element’s activity derives from low expression. These data demonstrate that even though spliced LINE-1 transcripts can complete retrotransposition, the resulting SpIREs are likely evolutionary dead ends, incapable of completing the next round of retrotransposition with any appreciable frequency.

As a testament to the power of studying the LINE-1 fossil record, Larson and coauthors provide a compelling case for the series of events that led to the birth of a new SpIRE. The authors utilize constructs generated by Jacobs and colleagues that contain or lack a 129-bp stretch of DNA shown to bind ZNF93, a restriction factor that initiates silencing of transcription (Fig 1) [18]. Elements without this sequence evade the repressive effects of ZNF93 binding, but deletion of this sequence also alters the spacing between the splice acceptor and splice donor sites of LINE-1. Larson et al. reasoned that this change in spacing should affect the length of any SpIREs produced from these evasive elements and could affect the efficiency of splicing and the subsequent replicative capacity of descendant SpIREs. In the absence of any inhibition from ZNF93, the authors find that LINE-1s with or without the 129-bp sequence are transcribed and retrotransposed at similar levels. However, analysis of the splice forms produced from these 2 LINE-1s shows that the deletion of the ZNF93 binding sequence alters the splicing pattern of the resulting LINE-1 sequences. Indeed, the new family of SpIREs described in this work comes specifically from LINE-1s with this deletion. Supportive of the very recent or even ongoing nature of this evolution, these recently formed SpIREs are highly polymorphic within the human population. These data reveal SpIREs as a new form of variation amongst humans.

In a very cool final experiment, the authors show that a construct that still contains the 129-bp ZNF93 binding sequence produces a previously undescribed SpIRE and that 9 copies of this new SpIRE can be found in the human genome. The authors reason that adaptation to host repression (the 129-bp deletion) should have altered the spliced forms of LINE-1. With these data, they demonstrate that these altered spliced transcripts are produced in vitro, and they (amazingly) show that indeed the in vitro analysis reflects what happened in nature because these variant splice forms can be found in the human genome.

In nature, successful reproduction requires propagation of the information necessary for replicating. The act of copying is alone insufficient (unless some utility is derived from that nonreproducing copy, like sterile worker ants): the new copies must be able to copy themselves. For selfish genetic elements, hosts encode specific mechanisms to block the replicative ability of descendant copies. These include proteins like APOBEC3s that deaminate intermediates in the replication of pathogens like LINE-1 and infectious viruses [27]. The new work from Larson and colleagues posits that splicing may be another mechanism of restriction of LINE-1 that could be employed in specific cell types or points in development (Fig 1). It would be interesting to expand this work to measure the presence and abundance of SpIREs in diverse cell types that have been previously shown to express variable sets of full-length LINE-1s [28].

The selection for deletion-containing LINE-1 sequences that evade ZNF93 seems to have altered the patterns and perhaps the frequency of splicing of these pathogens’ RNA. One could imagine that certain deletions would make LINE-1 more susceptible to repression by splicing. Alternatively, splicing could generate dramatic variation in the 5′-UTRs of these elements, some of which could have new functions; for example, some LINE-1 splice variants could delete sequences that the host targets to repress these elements, thereby creating a new escape variant of LINE-1. As always, this benefit would be weighed against the detriment of losing the beneficial aspect of this sequence, for example, transcription factor binding sites that increase expression. These studies of dead LINE-1 sequences in the human genome reveal the push and pull of sequence changes that provide both benefit and detriment. Future studies will advance the limits of our attempts to decode the mass of information written in genome sequences with the goal of better understanding the long-standing coevolution of LINE-1s and their hosts.

## Abbreviations

LINE-1: long interspersed element-1
ORF: open reading frame
SpIRE: spliced integrated retrotransposed element
UTR: untranslated region
